# Challenges in life sciences and health systems in the 21st century

**DOI:** 10.3325/cmj.2014.55.184

**Published:** 2014-06

**Authors:** Shlomo Melmed, Sandor G. Vari

**Affiliations:** Cedars-Sinai Medical Center, Los Angeles, CA, USA

Cedars-Sinai Medical Center (CSMC), established in 1902, is a non-profit, tertiary hospital and multi-specialty academic health science center located in Los Angeles, California, United States. Over 500 predoctoral and postdoctoral students, residents, and fellows participate in more than 60 graduate medical education and doctoral programs. CSMC focuses on biomedical research and technologically advanced medical education based on an interdisciplinary collaboration between physicians and translational investigators. CSMC expertise is especially prominent in cardiovascular, genetics, gene therapy, gastroenterology, neuroscience, immunology, surgery, organ transplantation, stem cells, biomedical imaging, and cancer with more than 800 research projects under way led by 230 Principal Investigators. US News & World Report’s rankings place Cedars-Sinai in 11 adult specialties in the top 18 Honor Roll of health centers in the USA.

Twelve years ago CSMC started to build a regional research organization in Central and Eastern Europe (CEE) and created the International Research and Innovation Management Program to implement Cross-Atlantic regional relationships in biomedical research for mutual benefits. In 2006 Cedars-Sinai Medical Center with eleven CEE universities and academic organizations from six countries (Croatia, Czech Republic, Hungary, Romania, Slovakia, and Ukraine) formed the Regional Cooperation for Health, Science and Technology (RECOOP HST) Consortium (1). In 2012, CSMC and the CEE partner organizations agreed to form the RECOOP HST Association and it is expected that the strategic partnership will add value to both CSMC and CEE.

Health care systems worldwide are facing similar challenges. The high gross national income (GNI) countries are experiencing problems of steadily increasing costs, unreasonable use of technologies, and decreasing consumer satisfaction. The lower middle and upper middle GNI countries face challenges related to quality of services and access to care (2,3). Rapidly advancing information technology provides unique opportunities to implement worldwide medical research collaborations to overcome challenges facing national health systems (4).

Cedars-Sinai is delighted to participate in this trans-continental initiative to enrich and enhance multi-community discovery in medicine. As a fully integrated clinical, research, education, and service enterprise, CSMC mission is to enhance the health care by employing cutting-edge scientific and information technologies (5). Accordingly, CSMC participation in RECOOP is certainly congruent with its mission, and an opportunity to share its expertise, and learn from other partners (2,4,5).

CSMC supports the training of graduate and post-graduate scientists in translational biomedical research, to assure the future professional manpower needs and to overcome the contests of complex scientific challenges. Globalizing this effort will foster healthy interchange of learning skills, as well as better education of the medical investigators of tomorrow (2). Furthermore, given the need for large-scale population and genomic data sets to accurately determine both disease etiology and individualized therapies, designing better health care requires collaborations which transcend both traditional institutions and countries (6,7).

RECOOP enables the opportunity for development of diverse talents, all geared toward integration of new knowledge derived from multispecialties in different countries. In particular it exploits this wonderful window for pursuing productive large-scale population studies. The highly talented and motivated RECOOP young trainees and established faculty, together with the Cedars-Sinai specialists, will successfully enable a strong multifaceted global approach to develop collaborations relying on the mutual strengths of so many gifted individuals. Production of new knowledge medicine is immeasurably facilitated by the enrichment opportunities provided by such a fluid and efficient trans-national scientific partnership and collaboration. This two-way partnership will immeasurably benefit both sides of the Atlantic in a mutual advancement of our global health mission.
